# Optimizing FRET-FLIM Labeling Conditions to Detect Nuclear Protein Interactions at Native Expression Levels in Living Arabidopsis Roots

**DOI:** 10.3389/fpls.2018.00639

**Published:** 2018-05-15

**Authors:** Yuchen Long, Yvonne Stahl, Stefanie Weidtkamp-Peters, Wouter Smet, Yujuan Du, Theodorus W. J. Gadella, Joachim Goedhart, Ben Scheres, Ikram Blilou

**Affiliations:** ^1^Plant Developmental Biology, Wageningen University and Research Centre, Wageningen, Netherlands; ^2^Institute for Developmental Genetics, Heinrich Heine University, Düsseldorf, Germany; ^3^Center for Advanced Imaging, Heinrich Heine University, Düsseldorf, Germany; ^4^Section of Molecular Cytology, van Leeuwenhoek Centre for Advanced Microscopy, Swammerdam Institute for Life Sciences, University of Amsterdam, Amsterdam, Netherlands; ^5^Plant Cell and Developmental Biology, King Abdullah University of Science and Technology (KAUST), Biological and Environmental Sciences and Engineering (BESE), Thuwal, Saudi Arabia

**Keywords:** protein complexes, protein-protein interaction, fluorescent proteins, *in vivo* FRET-FLIM, SHORT-ROOT, SCARECROW

## Abstract

Protein complex formation has been extensively studied using Förster resonance energy transfer (FRET) measured by Fluorescence Lifetime Imaging Microscopy (FLIM). However, implementing this technology to detect protein interactions in living multicellular organism at single-cell resolution and under native condition is still difficult to achieve. Here we describe the optimization of the labeling conditions to detect FRET-FLIM in living plants. This study exemplifies optimization procedure involving the identification of the optimal position for the labels either at the N or C terminal region and the selection of the bright and suitable, fluorescent proteins as donor and acceptor labels for the FRET study. With an effective optimization strategy, we were able to detect the interaction between the stem cell regulators SHORT-ROOT and SCARECROW at endogenous expression levels in the root pole of living Arabidopsis embryos and developing lateral roots by FRET-FLIM. Using this approach we show that the spatial profile of interaction between two transcription factors can be highly modulated in reoccurring and structurally resembling organs, thus providing new information on the dynamic redistribution of nuclear protein complex configurations in different developmental stages. In principle, our optimization procedure for transcription factor complexes is applicable to any biological system.

## Introduction

In living organisms, many cellular functions are executed by protein complexes. Over the decades, the concept of “protein-protein interaction networks” has emerged: rather than working as monomeric entities, most cellular proteins are known to dynamically engage in binding events. To understand the dynamic nature of these protein complexes, it is crucial to correlate the *in vivo* spatiotemporal interactions between key proteins and their impact on different biological processes. This holds true especially in a multicellular context, where heterogeneity of protein complexes between cell populations can lead to different outcomes in distinct cells within an intact organism.

Protein interactions are frequently studied with biochemical methods. These methods can be arduous, especially for protein complexes of low abundance. Improvements of protein purification procedures and the increased sensitivity of mass spectrometers have dramatically enhanced protein complex detectability (Bensimon et al., 2012; Pardo and Choudhary, 2012; Young et al., 2012; Aryal et al., 2014; Jorge et al., 2016; Wendrich et al., 2017). In addition, automated methods have been developed to isolate specific cell populations, further allowing high throughput proteome-wide analysis of protein complexes in selected cellular environments (Bridgeman et al., 2010; Petricka et al., 2012). Despite these technical advances, biochemical methods remain challenging when dealing with dynamic interactions in transient protein complexes.

**Figure 1:**
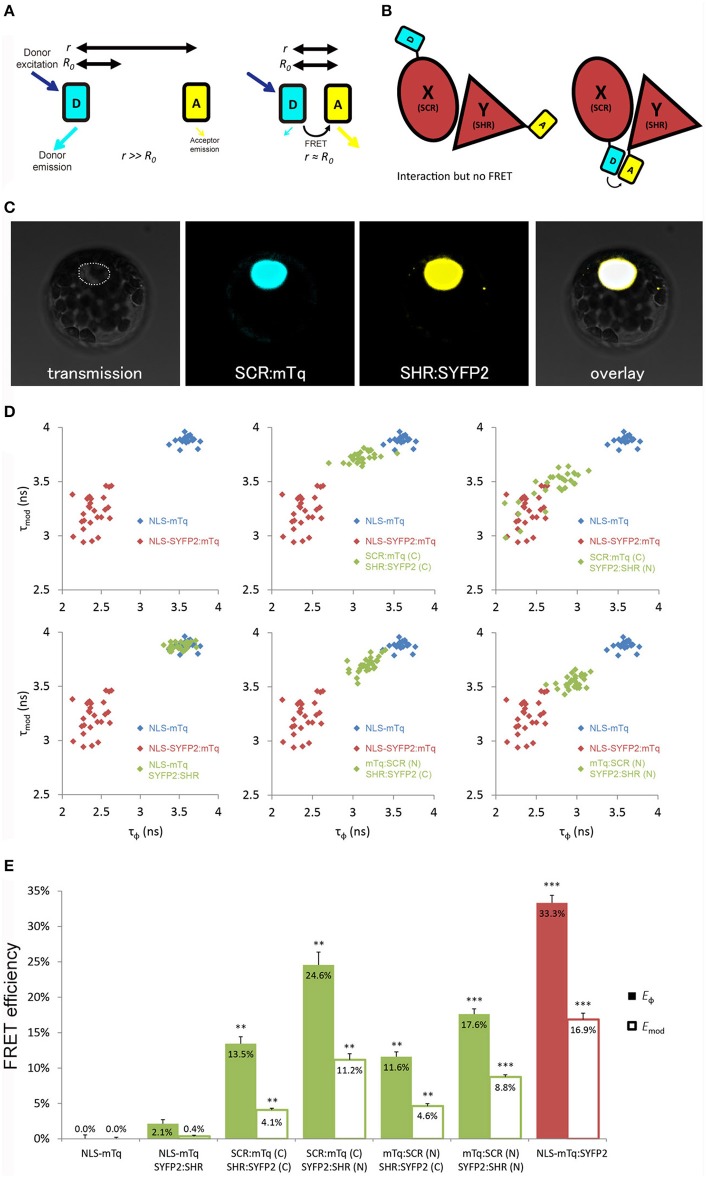
Optimization of tagging orientation for FRET-FLIM detection. **(A)** Illustration of FRET principle. D, donor fluorophore; A, acceptor fluorophore; *r*, distance between D and A; *R*_0_, Förster radius for D and A. **(B)** Illustration emphasizing the necessity to optimize tagging orientation for FRET. X and Y, two proteins of interest. Limited to no FRET might be observed when fluorophores are located at the distant ends of X and Y, yielding false negative result. **(C)** Arabidopsis mesophyll protoplast co-expressing SCR:mTq and SHR:SYFP2. Dotted line circles the nucleus. **(D)** Scatterplots showing distribution of phase lifetime τ_ϕ_ against modulation lifetime τ_*mod*_ from protoplast measurements. Each FRET pair was plotted against the same positive and donor-only samples. *n* > 10 for each sample. **(E)** Bar chart showing FRET efficiency *E* derived from τ_ϕ_ and τ_*mod*_ in **(D)**, error bars represent standard errors within one set of experiment. * represent *p*-values, **, 10^−20^ < *p* < 10^−2^; ***p < 10^−20^, *p*-values calculated by Student's *t*-test compared to the donor-only samples.

Alternatively, fluorescence-based microscopic techniques have been developed to study protein-protein interactions. Bimolecular fluorescence complementation (BiFC) assays are commonly employed to visualize protein interaction in living cells, where two non-fluorescent fragments of a fluorescent protein can form a bimolecular fluorescent complex upon interaction (Hu et al., 2002). Successful BiFC applications in intact living organisms have been reported (Zhang et al., 2004; Gohl et al., 2010; Hudry et al., 2011; Smaczniak et al., 2012). However, the irreversible formation of bimolecular complexes limits its use to follow dynamic protein interactions (Lalonde et al., 2008; Horstman et al., 2014; Xing et al., 2016). Conversely, other strategies such as employing Förster resonance energy transfer (FRET) can provide better means to visualize and quantify dynamic protein complexes in living cells (Weidtkamp-Peters and Stahl, 2017), with the spatial information presented as a microscopic lifetime image. FRET describes the phenomenon of energy transfer from an excited donor fluorophore to a non-excited acceptor chromophore in its direct vicinity through dipole-dipole coupling (Förster, 1948; Figure 1A). Since FRET only occurs when the two fluorophores are within a short radius (on the scale of several nanometers), direct protein-protein interaction can be detected by tagging candidate proteins with appropriate fluorophores, such as different green fluorescent protein (GFP) variants (Kremers and Goedhart, 2009). Upon interaction, FRET will lead to a decreased donor emission, relative to that measured in a non-FRET situation, and an elevated acceptor emission (Clegg, 2009). These changes in emission intensities can be used to reflect the level of protein interaction by directly monitoring donor-acceptor emission ratio changes or measuring donor emission recovery after acceptor photobleaching (Gu et al., 2004; Adjobo-Hermans et al., 2011). However, these emission level-based techniques are highly dependent on the concentrations and good signal-to-noise ratios of both donor and acceptor, which are often difficult to achieve for lowly expressed proteins at endogenous levels.

FRET can also be quantified by measuring the fluorescence lifetime decrease of the donor molecules by fluorescence lifetime imaging microscopy (FLIM) (Gadella et al., 1993). Applications of FRET-FLIM have been mostly applied to analyze protein-protein interaction in living cells or as means to analyze biosensors (Tonaco et al., 2006; Crosby et al., 2011; Kardash et al., 2011; Bücherl et al., 2013; Stahl et al., 2013). Since accurate FRET-FLIM measurements are less dependent on emission intensity, it can be especially useful to detect interaction between proteins under native conditions without resorting to overexpression, which can alter cell states. Therefore, dynamic protein complex association at cellular resolution can be detected non-invasively using a microscopy-based approach (Bücherl et al., 2013). With these technical advantages, one would be able to follow and quantify such interactions in living multicellular organisms and determine their specificity in different cell types and developmental contexts.

Recently we have shown that FRET-FLIM can be used to study transcription factor associations in the model organism *Arabidopsis thaliana* (Long et al., 2017), particularly in the root tip which is ideal for live imaging with confocal microscopy due to its transparency and its simple, organized structure. We exploited the intensively-studied interaction between the two GRAS domain transcription factors SHORT-ROOT (SHR) and SCARECROW (SCR) (Di Laurenzio et al., 1996; Helariutta et al., 2000). SHR and SCR control the radial pattern of the Arabidopsis root through generating formative cell divisions in the stem cell called the cortex-endodermis initial (CEI) (Di Laurenzio et al., 1996; Helariutta et al., 2000). SHR is also required for endodermal specification (Helariutta et al., 2000; Long et al., 2015a,b; Moreno-Risueno et al., 2015). *SHR* transcript is produced in the vasculature and its protein moves outward into the surrounding cell layer consisting the quiescent center (QC), CEI and endodermis, collectively called as the U-shaped domain (Nakajima et al., 2001; Supplementary Figure 1). SHR physically interacts with SCR in the U-shaped domain of the main root, and the interaction is more pronounced in the CEI to regulate downstream target expressions such as *CYCLIN D6;1* (*CYCD6;1*) to promote formative divisions (Cui et al., 2007; Cruz-Ramírez et al., 2012; Long et al., 2015a, 2017).

Here, we provide a guideline for utilizing the FRET-FLIM technology to visualize and quantify protein interactions at physiological conditions in living Arabidopsis roots at cellular resolution, with SCR and SHR as the example protein pair. We extended our analysis to Arabidopsis lateral roots and embryos to show that *in vivo* FRET-FLIM can also be applied to visualize interactions in other organs. In this study, we addressed the key optimization steps for transcription factors as prerequisites for measurable FRET to occur in living Arabidopsis tissues. These include (1) testing position of fluorescent tags at amino- and carboxyl-termini of each tested protein, (2) evaluating fluorophores suitability, and (3) *in vivo* fusion protein functionality.

Our work demonstrates that *in vivo* FRET-FLIM can be used to visualize nuclear protein interactions in a living, intact organism and provides evidence that the level of interaction between transcription factors can be heterogeneous throughout their domain of co-localization, and their interaction pattern can change depending on the developmental stage. Our optimization set up and procedure to detect *in vivo* protein-protein interaction can in principle be applied to any protein pair in any biological system.

## Materials and methods

### DNA constructs

Coding sequences (CDS) of *SCFP3A, mTurquoise, SYFP2, mCherry, mStrawberry* and *mRFP* (Kremers et al., 2006; Goedhart et al., 2007) were subcloned into multiple Gateway cassettes with flanking *att*B sites. A general SV40 nuclear localizing signal (NLS) (Lassner et al., 1991) was attached to the N-terminal of *mTq* and *SYFP2* to generate *NLS-mTq* and *NLS-SYFP2*. For C-terminal tagging, fluorescent protein sequences were recombined into pGEMTeasyR2R3 vector by Gateway BP reaction; while pGEMTeasyR1R2-derived entry clones were generated for N-terminal tagging. *SHR* and *SCR* coding sequence in pDONR221-derived entry clones (Welch et al., 2007) were used for C-terminal tagging clones; while for N-terminal tagging *SHR* and *SCR* were subcloned into pGEMTeasyR2R3. For protoplast transfection, *35S* promoter-driven fusions of *SHR* and *SCR* with N- or C-terminal tagging were created in pB7m34GW or pH7m34GW binary vectors (Karimi et al., 2007) by multiple Gateway LR reactions (Invitrogen). Positive controls of *35S::NLS-SYFP2:mTq* and *35S::NLS-SYFP2:SCFP3A* were generated by combining previously described tags in entry clones. Root expression vectors of *SHR* and *SCR* were created similarly with endogenous *pSHR* and *pSCR* promoters (Long et al., 2015a). For better stem cell niche localization (see below), *pSHR::SYFP2-SHR*Δ*1a* was generated by site-directed mutagenesis (QuikChange II, Aligent) from *pSHR::SYFP2:SHR* vector. For HeLa cell expression, SYFP-Δ1a-SHR was generated by subcloning *SHR* CDS with flanking restriction sites (5′-BsrGI-SHR-BamHI-3′) into pSYFP2-C1 (Kremers et al., 2006) followed by site-directed mutagenesis as described. SCR-mCherry was generated by subcloning *SCR* CDS with flanking restriction sites (5′-KpnI-SCR-AgeI-3′) into pmTurquoise-N1 (Goedhart et al., 2010), followed by swapping *mTurquoise* with *mCherry* (5′-AgeI-mCherry-NotI-3′) (Goedhart et al., 2007). Primers for cloning are listed in Supplementary Table 1.

### Arabidopsis growth condition and transformation

*Arabidopsis thaliana* ecotype Columbia (Col-0) plants containing *SHR* and *SCR* transgenes were grown as previously described (Long et al., 2017). Stably transformed lines were generated by *Agrobacterium tumefaciens*-mediated transformation via floral dip method (Clough and Bent, 1998).

### Protoplast preparation and transfection

*A. thaliana* Col-0 mesophyll protoplasts were prepared and transfected according to (Díaz-Triviño et al. (2017). *A. thaliana* Col-0 tissue culture protoplasts were prepared and transfected according to Axelos et al. (1992). Ten microgram donor vector and 20 μg acceptor vector were transfected.

### Transfection of heterologous systems

HeLa cell culture and transfection were as described in Jiang et al. (2014), constructs were transfected using FuGENE 6 protocol (Promega).

### Fluorescence lifetime imaging microscopy in protoplasts

Living transfected protoplasts were collected in LabTek chambered coverglass (Nunc) for frequency-domain FLIM measurements. Samples with cyan fluorescent donors were acquired according to Goedhart et al. (2010) and samples with yellow fluorescent donor were acquired according to Goedhart et al. (2007). Briefly, CFP-variants were excited with a 440 nm modulated diode laser (LDH-M-C-440; PicoQuant) at 75.1 MHz, the light was reflected by a 455DCLP dichroic mirror and emission was passed through a D480/40 band-pass emission filter (Chroma Technology). SYFP2 fluorescence was excited with a 514 nm Argon laser (Melles-Griot) intensity-modulated at a frequency of 75.1 MHz and the light was reflected by a 525DCXR dichroic mirror and emission was passed through a HQ545/30 band-pass emission filter (Chroma Technology). Emission was detected using a radio frequency (RF)-modulated image intensifier (Lambert Instruments II18MD) coupled to a charge-coupled device (CCD) camera (Photometrics HQ) as detector. FLIM stacks of 18 phase images were acquired in permutated recording order with an exposure time of 50-1000 ms per image depending on sample brightness. The average fluorescence lifetime of individual nuclei was quantified from which an average lifetime for the sample was determined. FRET efficiency was calculated as described in Goedhart et al. (2007) More than 10 cells were analyzed for each sample.

### Confocal microscopy

Protoplasts, Arabidopsis embryos and lateral roots were imaged with a LSM 710 laser-scanning confocal microscope (Carl Zeiss GmbH) with an Objective C-Apochromat 40x/1.2 W Corr M27. A 2 air unit (AU) pinhole was set for weak *SHR* expression. Cyan fluorescence was detected at 465–500 nm with 458 nm excitation and 458/514 beam splitter; yellow detected at 520–560 nm with 514 nm excitation and 458/514 beam splitter; and red detected at 600–660 nm with 543 nm excitation and 488/543/633 beam splitter, respectively. Images were taken with no offset, and signal-to-noise ratio (SNR) was calculated as follows:

(1) $$\begin{array}{r} \text{SNR}=\frac{S}{N} \end{array}$$

where *S* is the nuclear fluorescence signal from imaged root endodermis, and *N* auto-fluorescence signal in the adjacent non-fluorescent area in the root to emphasize the challenge of measurement in Arabidopsis root with high background signal. More than 10 roots were analyzed for each SNR calculation, except for *pSCR::SCR:mStrawberry* (*n* = 8), *pSCR::SCR:mCherry* (*n* = 7) and *pSHR::SHR:mRFP* (*n* = 9).

### Fluorescence lifetime imaging microscopy in living arabidopsis

Roots of 6 dpg seedlings were mounted in water for measurements in LRP. Late heart-/early torpedo-stage embryos were mounted in 5% glucose for measurements. FLIM was performed on a confocal laser scanning microscope (Zeiss LSM 780) additionally equipped with a single-photon counting device with picosecond time resolution (PicoQuant Hydra Harp 400). SYFP2 fluorescence was excited at 485 nm using a linearly polarized diode laser (LDH-D-C-485) operated at a repetition rate of 32 MHz. Excitation power was around 1 μW at the objective C-Apochromat 40x/1.2 W Corr M27). The emitted light was collected in the same objective and separated into its perpendicular and parallel polarization (Thorlabs PBS 101, Thorlabs GmbH, Germany). Fluorescence was then detected by Tau-SPADs (PicoQuant) in a narrow range of SYFP2's emission spectrum (band-pass filter: HC535/30 AHF). Images were taken with 12.6 μs pixel time and a resolution of 0.1 μm/pixel for roots and embryos and 0.21 μm/pixel for LRP (Zoom 4 and 2, 256 × 256). A series of 60 frames were merged into one image and further analyzed (Widengren et al., 2006).

### Single-pixel fluorescence lifetime analysis

The fluorescence lifetime of SYFP2 was determined and analyzed pixel-wise in merged images to increase photon numbers for analysis using the software tools “AnI-3SF” and “Margarita” developed in Prof. C.A.M Seidel group [Software Package for Multiparameter Fluorescence Spectroscopy, Full Correlation and Multiparameter Fluorescence Imaging (http://www.mpc.uni-duesseldorf.de/en/software/software-package.html)] for Multiparameter Fluorescence Image Spectroscopy (MFIS) (Kudryavtsev et al., 2007; Weidtkamp-Peters et al., 2009). In fluorescence lifetime measurements, high spatial resolution microscopy and low excitation power prevent photo bleaching; the number of photons per pixel is exceptionally low, ranging from 100 to 2,000 photons per pixel. Therefore, a model to fit the data with a minimal number of parameters has to be applied in conjunction with a maximum likelihood estimator (MLE) (Schaffer et al., 1999; Eggeling et al., 2001; Widengren et al., 2006; Weidtkamp-Peters et al., 2009; Sisamakis et al., 2010). The decay of SYFP2 is approximated in the subsequent fluorescence lifetime analysis by an (fluorescence-weighted) average lifetime, τ. We therefore used a monoexponential model function with two variables (fluorescence lifetime τ and scatter contribution γ); as described elsewhere (Stahl et al., 2013), fitted with MLE. The instrument response function was measured using the dye erythrosine, which exhibits a very short fluorescence lifetime, which is additionally quenched in an aqueous, saturated potassium iodide solution.

### FRET-FLIM quantification in living arabidopsis

Nuclear areas of no smaller than 25 pixels, based on the nuclei's appearances after the 100-photon-per-pixel background subtraction, were selected from independent cells. Cellular fluorescence lifetimes were computed by least-square fitting the Gaussian peaks of each cells' lifetime distributions. Fluorescence lifetimes at the same cell position were pooled from independent measurements without normalization, enabled by the robust FRET-FLIM acquisition between samples and between experiments. Reduction of fluorescence lifetime (Δτ) between donor-only and FRET samples were calculated from the means of donor-only and FRET samples at each cell position, with inclusion of fractional standard errors. Significances, between donor-only and FRET samples at specific cell positions in the same or different experiments, were resolved by Student's *t*-test with critical value of *p* < 0.01.

## Results

### Experimental design for *in vivo* FRET-FLIM optimization

Our optimization procedure featured an *ex vivo* to *in vivo* pipeline, where we first employed the transient Arabidopsis protoplast expression system as a convenient tool to test a large number of FRET-FLIM pair combinations to select optimal positions of fluorescent tags and system-specific fluorophores, before evaluating protein functionality in Arabidopsis roots. For rapid data acquisition, we exploited widefield frequency-domain FLIM (Supplementary Figure 2A; Verveer and Hanley, 2009) measurements for protoplast samples with high transgene expression levels. Lifetime measurements in living Arabidopsis tissues were conducted with time-correlated single photon counting (TCSPC)-based time-domain FLIM (Supplementary Figure 2B; Gerritsen et al., 2009) with confocal imaging of lowly-expressed proteins at endogenous levels.

### Position of fluorescent tags

Close proximity between the donor and the acceptor is a prerequisite for achieving measureable FRET (Figure 1B). We first optimized the tagging position to detect FRET between SHR and SCR with a cyan-emitting mTurquoise (mTq) (Goedhart et al., 2010) as donor and a yellow-emitting SYFP2 (Kremers et al., 2006) as acceptor in Arabidopsis protoplasts. We fused mTq and SYFP2 to either the amino- or carboxyl-termini of the SHR and SCR proteins. We constructed *SCR:mTq, mTq:SCR, SHR:SYFP2*, and *SYFP2:SHR* under the constitutive promoter of Cauliflower Mosaic Virus 35S RNA (*35S*) by the Gateway cloning system, and introduced them into Arabidopsis protoplasts as pairs (example in Figure 1C). As a negative control, we co-transfected *SYFP2:SHR* with a nuclear-localizing mTq (*NLS-mTq*), while for positive control we constructed a nuclear-localizing fusion between SYFP2 and mTq (*NLS-SYFP2:mTq*), where constitutive FRET occurs. Upon paired co-transfection, we measured lifetimes for each SHR-SCR combination by frequency-domain FLIM measurements. Frequency domain FLIM measurements yield a fluorescence lifetime based on the phase shift (τ_ϕ_) and demodulation (τ_*mod*_) of the fluorescence emission relative to the modulated excitation source (Supplementary Figure 2A; Verveer and Hanley, 2009). From these lifetimes and the lifetime of the donor-only sample, the average FRET efficiency was calculated, yielding *E*_ϕ_ and *E*_*mod*_ (Supplementary Figure 2A). As shown in Figures 1D,E, different combinations of tagging orientations gave varying levels of lifetime changes, i.e., different shifts of lifetimes in the scatterplots. This results in the unequal FRET efficiencies in the bar chart. The SCR:mTq SYFP2:SHR combination scored the highest FRET efficiency of *E*_ϕ_ = 24.6% ± 1.8% and *E*_*mod*_ = 11.2% ± 0.9% (Figures 1D,E; Long et al., 2017). These results suggest that the carboxyl-terminus of SCR and the amino-terminus of SHR are in close proximity. Up to 33.3% FRET efficiency was measured in the positive control NLS-SYFP2::mTq (Figure 1E), comparable to the previous reported value (Goedhart et al., 2010). The NLS-mTq SYFP2:SHR negative control gave near-ground level FRET (Figure 1E), indicating that FRET between each SHR-SCR combination reflects specific binding. To achieve the highest sensitivity, we selected carboxyl-terminal-tagged SCR and amino-terminal-tagged SHR for further optimizations and analyses.

### Suitability of the fluorophores

The brightness and quantum yield of the fluorescent proteins depends on pH, temperature and other conditions introduced by different biological systems. To identify the optimal fluorophores suitable for FRET-FLIM measurement in Arabidopsis, we compared the performances of several fluorescent proteins in protoplasts and roots (Tables 1, 2).

**Table 1:** Summary of fluorophores used in this study and their performance in transient systems.

**Fluorophore**	**Expression under constitutive promoters in transient systems**	**FRET efficiency**	**Recommended for TF expression and FLIM experiments**
SCFP3A	High	Good	+++
mTurquoise	High	Very good	+++
SYFP2	High	Good	+++
mCherry	Moderate	Good	+++
mStrawberry	Moderate	Moderate	++
mRFP	High	Good	+++

**Table 2:** Overview on the fluorophores performance when used under native promotors in living Arabidopsis roots.

**Fluorophore**	***In vivo* expression under endogenous or tissue specific promoters**	***In vivo* signal-to-noise ratio**	**Suitable for TF expression and FLIM experiments**
SCFP3A	Low	Moderate	(Not suitable because of high background)
mTurquoise	Very low to not detectable	Low	(Not suitable because of low detectability)
SYFP2	High	Good	+
mCherry	Very low	Low	(Not suitable because of low detectability)
mStrawberry	Very low	Low	(Not suitable because of low detectability)
mRFP	High	Good^*^	+

* *The intensity is depending on the promoter activity, while for SCR promoter the levels were suitable for this study, mRFP intensity is too low for SHR as an acceptor under its endogenous promoter*.

**Figure 2:**
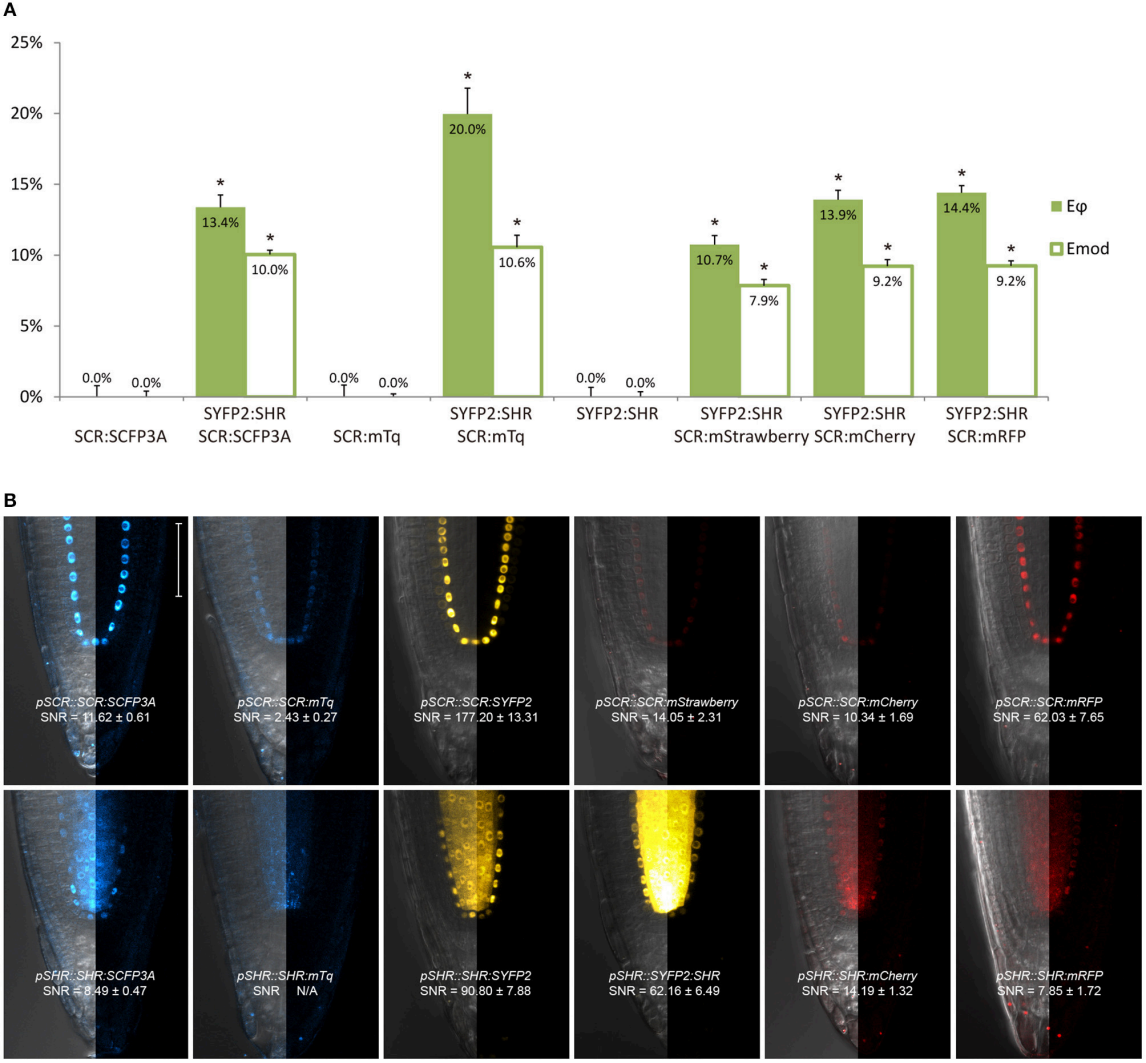
Selection of an appropriate fluorescent protein pair for FRET-FLIM analysis. **(A)** Bar chart of FRET efficiency *E*_ϕ_ and *E*_*mod*_ between SCR and SHR tagged with different fluorescent proteins, with error bars of standard error of mean, *n* > 10 for each sample. *p < 10^−2^, *p*-values calculated by Student's *t*-test compared to the donor-only samples. **(B)** Confocal images of roots expressing SCR and SHR tagged with different fluorescent proteins, with signal-to-noise ratio (SNR) calculated from endodermal nuclear fluorescence signal. Scale bar, 50 μm. Each image displays the overlay image of transmission and fluorescent channels in the left half and the fluorescence channel in the right half from the same root.

First, we evaluated whether cyan fluorescent protein (CFP) variants SCFP3A and mTq, in the context of our FRET pair combination SCR and SHR, could be used in a common cyan-yellow FRET-FLIM setup in plant cells (Kremers et al., 2006; Hamers et al., 2014). As shown in Figure 2A, SCR:mTq yielded a higher FRET efficiency than SCR:SCFP3A in combination with SYFP2:SHR in protoplasts, most likely due to mTq's higher quantum yield. However, SCR:SCFP3A SYFP2:SHR measurements were more precise (Figure 2A, Supplementary Figure 3A). The reduced precision of mTq-SYFP2 measurements might reflect suboptimal mTq performance in plant nuclei (see Discussion).

We next tested the performance of SCFP3A, mTq and SYFP2 in Arabidopsis roots. Since SHR and SCR co-localize in the U-shaped domain, it is essential to detect them in these cells to assess where they interact. Under endogenous promoters, both cyan-variant-tagged *SCR* and *SHR* transgenic lines displayed low fluorescence levels relative to the background: signal of *pSCR::SCR:SCFP3A, pSCR::SCR:mTq*, and *pSHR::SHR:SCFP3A* could be detected in the endodermis with low signal-to-noise ratios (SNR); while endodermal signal of *pSHR::SHR:mTq* was indistinguishable from background signal (Figure 2B).

Since FRET-FLIM is more dependent on donor fluorescence, the poor detection of these two cyan variants made them unsuitable as donor tags in this system. On the contrary, *pSCR::SCR:SYFP2* and *pSHR::SYFP2:SHR* yielded readily detectable emissions supported by higher SNR (Figure 2B), hence we favored SYFP2 as donor tag. Since it has been previously shown that red fluorescent proteins are efficient FRET acceptors for SYFP2 with Förster radii > 5.6 nm (Goedhart et al., 2007), we proceeded to optimize the labeling conditions for yellow-red FRET pairs.

Three red-emitting variants, mStrawberry, mCherry and mRFP, were tested for their performance as mentioned above. In protoplasts, SHR and SCR tagged with all three red variants and SYFP2 gave comparable FRET efficiency, with SYFP2-mStrawberry pair slightly lower (Figure 2A, Supplementary Figure 3B). When expressed in roots, *pSCR::SCR:mRFP* exhibited higher detectability than *pSCR::SCR:mStrawberry* and *pSCR::SCR:mCherry*, making mRFP a better choice. In the case of SHR, all the red variants displayed low detectability correlating with low signal-to-noise ratios (SNR) (Figure 2B). Considering that sufficient FRET analysis requires more acceptor molecules than donors, or “donor saturation,” SCR is then more suitable as acceptor due to its higher endogenous expression level than SHR (Long et al., 2017, Table 3). Therefore, we selected SYFP2:SHR and SCR:mRFP for *in vivo* FRET-FLIM studies.

**Table 3:** Summary of the performance of fluorophore pairs used in this study.

**FRET pair**	**Suitability for FRET-FLIM in transient systems**	Suitability for native FRET-FLIM^*^
SCFP3A—SYFP2	+++	–
mTurquoise—SYFP2	++	–
SYFP2—mCherry	++	–
SYFP2—mStrawberry	++	–
SYFP2—mRFP	+++	+

* *Note that this is strictly dependent on the level of expression and the stability of the protein of interest*.

### *in vivo* fusion protein functionality

Tagging proteins of interest with fluorescent proteins has a potential pitfall: the resulting fusions might reduce biological function due to undesired conformational changes or steric hindrance introduced by the tags. Measurements carried out with such non-functional or dysfunctional fusions might not accurately reflect their endogenous behaviors. Therefore, it is crucial to evaluate the functionality of fusion proteins before FRET-FLIM measurements.

**Figure 3:**
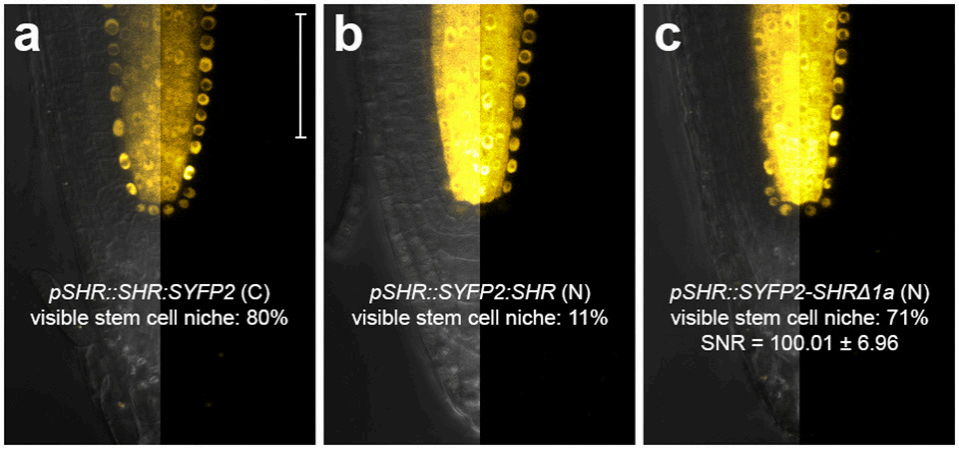
Improvement of SHR fusion protein mobility. Confocal images of roots expressing SHR fusion proteins differentially tagged with SYFP2, with signal-to-noise ratio (SNR) calculated from endodermal nuclear fluorescence signal. **(a)** *pSHR::SHR:SYFP2*, **(b)** *pSHR::SYFP2:SHR*, **(c)** *pSHR::SYFP2-SHR*Δ*1a, n* > 10 for each sample. Scale bar, 50 μm. For every image, the left half displays the overlay image and the right half fluorescence channel from the same root.

The C terminal fusion *pSCR::SCR:mRFP* was reported to be functional (Long et al., 2015a, 2017). For SHR fusion, despite its high detectability in the endodermis, we noticed that only 11% of the roots harboring *pSHR::SYFP2:SHR* showed clearly visible signal in the stem cell niche (Figure 3b), while such signal was readily visible in 80% of roots harboring the carboxyl-terminal-tagged *pSHR::SHR:SYFP2* (Figure 3a). This indicated that SYFP2:SHR might not move efficiently between certain cells. As previously shown, SHR movement from the vasculature is essential for root growth regulation, and altering its mobility can cause abnormal CEI division and disrupted root architecture (Cui et al., 2007; Vatén et al., 2011; Koizumi et al., 2012; Long et al., 2015a). Additionally, SHR and SCR co-localize in the endodermis and stem cell niche, it is thus essential to have sufficient SHR movement into the stem cell niche to measure SHR-SCR interaction. Since amino-terminal tagging on SHR was not reported to disrupt SHR movement (Heidstra et al., 2004), we reasoned that the Gateway linker between SYFP2 and SHR might cause an undesired conformational change to the fusion, and attempted to restore SYFP2:SHR mobility by linker alteration. A typical *att*B2 Gateway recombination site with flanking sequence is recommended to be 27 base pairs after recombination (Invitrogen), translating to a linker of 9 amino acids DPAFLYKVA between SYFP2 and SHR. Although longer, more flexible linkers are usually favored for functional tagging, farther tag displacement can potentially increase the distance and reduce the probability of spatial association between donor and acceptor fluorophores beyond the Förster radii, thereby reducing FRET. Thus, we shortened the linker using site-directed mutagenesis, and generated *pSHR::SYFP2-SHR*Δ*1a* by removing 5 amino acids, reducing it from DPAFLYKVA to DKVA, similar in length to the described functional N-terminal SHR fusion (Heidstra et al., 2004). Both linkers are estimated to be shorter than the 5.6 nm Förster radius for SYFP2-mRFP pair (Goedhart et al., 2007). As shown in Figure 3c, up to 71% of the roots harboring *pSHR::SYFP2-SHR*Δ*1a* showed significant improvement of SHR fusion signal in the stem cell niche. The linker alteration of SYFP2-SHRΔ1a did not change the FRET efficiency between SHR and SCR in protoplasts (Supplementary Figures 3A,B), indicating that neither fluorophore distance nor dipole orientation was disrupted. This enabled us to measure FRET-FLIM between SHR and SCR in their endogenous conditions.

Our optimization procedure revealed that the combination of analysis in protoplasts (*ex vivo*) and intact plants (*in vivo*) is essential for the selection of the appropriate donor-acceptor pairs and protein fusions strategies for *in vivo* FRET-FLIM measurements. A summary of choosing the optimal fluorophores *ex vivo* and *in vivo* as well as additional considerations of using this technology can be found in Supplementary Materials.

### *in vivo* FRET-FLIM in different developmental contexts

In a previous study, we implemented *in vivo* FRET-FLIM measurements between SYFP2-SHRΔ1a and SCR:mRFP in the Arabidopsis primary root meristem, and showed that SHR and SCR interact in the QC, CEI and endodermis in Arabidopsis roots (Long et al., 2017). The primary root meristem is pre-established in the embryotic root pole (ten Hove et al., 2015), while *de novo* root meristems repetitively emerge in the forms of lateral roots, adventitious roots and during root regeneration (Verstraeten et al., 2014; Efroni et al., 2016). Although highly resembling in structure and sharing the transcriptional regulatory network, the precise regulatory mechanisms have been proposed to differ between these root meristems (Lucas et al., 2011; Verstraeten et al., 2014; Efroni et al., 2016; Du and Scheres, 2017). To explore the SHR-SCR interaction profile in other developmental contexts, we extend the application of *in vivo* FRET-FLIM measurements to Arabidopsis embryos and developing lateral roots.

**Figure 4:**
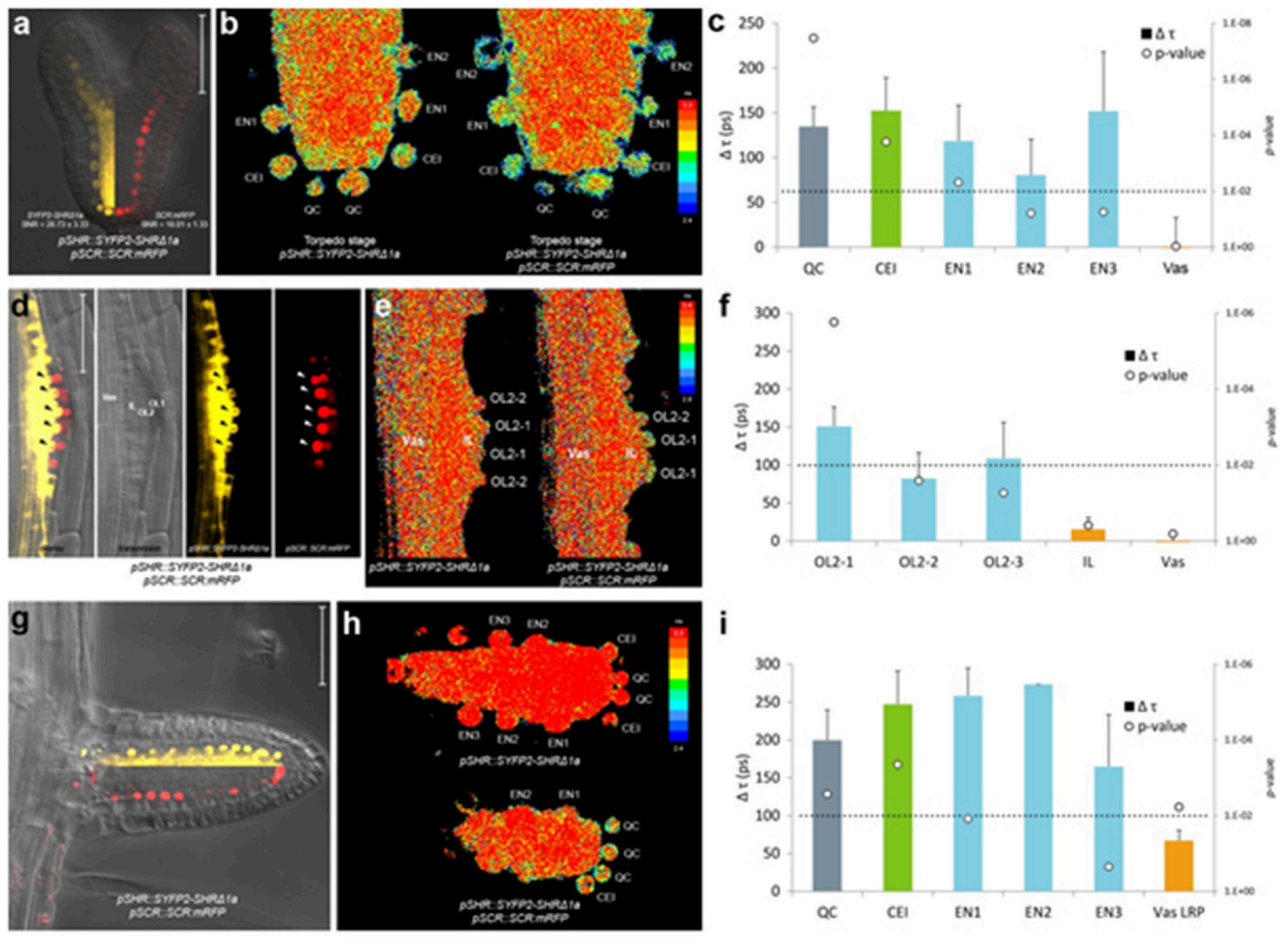
*In vivo* FRET-FLIM of SHR-SCR in embryos and lateral roots. **(a)** Early torpedo stage Arabidopsis embryo co-expressing *pSHR::SYFP2-SHR*Δ*1a* and *pSCR::SCR:mRFP*, with signal-to-noise ratio (SNR) calculated from endodermal nuclear fluorescence signal. Scale bar, 50 μm. Yellow fluorescence channel (left) and red fluorescence channel (right) were overlaid with transmission image from the same root. **(b)** Heatmaps of fluorescence lifetime in donor-only and sample embryo. **(c)** Quantification of lifetime change (Δτ) in single cells. Column color matches with tissue type illustrated in this figure. Circles indicate *p*-value calculated by Student's *t*-test of sample lifetimes comparing to donor-only lifetimes at each cell position, with the dotted line marking the 0.01 significant value. Donor embryos *n* = 18, FRET sample embryos *n* = 34. **(d)** Arabidopsis stage IV LRP co-expressing *pSHR::SYFP2-SHR*Δ*1a* and *pSCR::SCR:mRFP*. Scale bar, 50 μm. OL1 and OL2, outer layer 1 and 2; IL, inner layer; Vas, primary root vasculature. Arrowheads point to OL2 cells where SHR and SCR co-localize. **(e)** Fluorescence lifetime heatmaps of donor-only and sample LRP. OL2 cells were numbered with OL2-1 in the middle of the LRP and OL2-2 and−3 progressively further from LRP midline. **(f)** Quantification of FRET between SYFP2-SHRΔ1a and SCR:mRFP measured in **(e)**. Donor LRP *n* = 13, FRET sample LRP n = 17. **(g)** Arabidopsis emerged lateral root co-expressing *pSHR::SYFP2-SHR*Δ*1a* and *pSCR::SCR:mRFP*. Scale bar, 50 μm. Yellow fluorescence channel (upper) and red fluorescence channel (lower) were overlaid with transmission image from the same root. **(h)** Fluorescence lifetime heatmaps of donor-only and sample emerged lateral root. **(i)** Quantification of FRET between SYFP2-SHRΔ1a and SCR:mRFP measured in **(h)**. Donor lateral roots *n* = 11, FRET sample lateral roots *n* = 3. Vas LRP, vasculature of LRP.

In heart stage embryos, SHR and SCR expression domains at the root pole resemble those in the postembryonic roots (Figure 4a). Similar to the observations in the primary root meristem (Long et al., 2017), we found that SYFP2-SHRΔ1a exhibited strong FRET with SCR:mRFP in QC, CEI and endodermis of late heart-/early torpedo-stage embryos (Figures 4b,c). Interestingly, FRET between SYFP2-SHRΔ1a and SCR:mRFP in the embryo was enhanced in QC and the first endodermal cell (endodermis 1), to similar levels occurring in the CEI (Figure 4c). This observation might reflect enhanced SHR-SCR interaction or closer SHR-SCR association in multimeric protein complexes in these embryonic cells. Alternatively, the contribution of high background signal (reduced SNR in Figure 4a) with generally shorter lifetimes in the embryos might have influenced FRET detections and resulted in a general lifetime reduction. To distinguish between these possibilities, detailed expression analysis of direct target genes of SHR-SCR complex like *CYCD6;1* during embryogenesis, as well as creating mutations in the SHR-SCR interaction domain, will be necessary to fully understand these observations. Nevertheless, our *in vivo* FRET-FLIM results hint that, despite the structural resemblance and developmental similarity, the underlying molecular wiring regulating embryonic root can be different from the root tip.

New root meristems are formed from differentiated root tissue in a process called lateral root formation. Lateral root primordia (LRP) initiation is marked by a series of cell divisions originating from the vasculature, particularly the pericycle cells opposing the xylem pole (Malamy and Benfey, 1997). Using *in vivo* FRET-FLIM, we studied the interaction between SHR and SCR during lateral root formation. As shown in Figure 4d, SHR and SCR only co-localized in a subset of cells in the developing stage IV LRP: SCR:mRFP was detected in both of the two outer layers (OL1 and OL2), while SYFP2-SHRΔ1a resided in the OL2 nuclei and maintained nuclear-and-cytoplasmic localization in the inner layer (IL), similar to mature vasculature. Within OL2 where SYFP2-SHRΔ1a and SCR:mRFP co-localized, FRET was detected higher in the central cells (OL2-1, Figure 4e,f). In contrast, OL2 cells displaced from LRP midline (OL2-2 and OL2-3, Figure 4f) exhibited lower FRET levels similar to those in the endodermis in the primary root (Long et al., 2017). No FRET was detected in the IL or vasculature due to the absence of detectable SCR:mRFP (Figure 4f).

After emergence, the lateral root morphology resembles the primary root, with similar cellular organization and expression patterns of *SHR* and *SCR* (Figure 4g). However, the FRET levels between SYFP2-SHRΔ1a and SCR:mRFP in emerged lateral roots were generally higher with no significant difference between QC, CEI and endodermis (Figure 4i).

Analyses between SYFP2-SHRΔ1a and SCR:mRFP in Arabidopsis embryos and LRP show that *in vivo* FRET-FLIM can be utilized within different developmental contexts. The generally preserved but slightly altered interaction patterns further suggests that the transcriptional regulations of SHR and SCR may exhibit different network topology in different developmental stages.

### FRET-FLIM of plant proteins in heterologous system

Interaction between SHR and SCR has been shown by many approaches including assays in mammalian cells (Long et al., 2017). To assess whether this interaction can be detected by FRET-FLIM in a system devoided from plant specific transcriptional regulations, we measured FRET-FLIM between SYFP2-SHRΔ1a and SCR-mRFP the HeLa cells and we could detect interaction (Supplementary Figures 4C,D), albeit at a lower level. This demonstrates that plant protein interaction can be analyzed in heterologous systems like animal cells.

## Discussion

In the present study, we outline an optimization procedure of the labeling conditions for applying the FRET-FLIM technology to inspect nuclear protein interactions in living plants. We show that protein complex formation can be mapped to specific cells in different organs *in vivo* and that the interaction domain is spatially modulated during development. This technique therefore overcomes previous limitations to studying protein complex dynamics at cellular resolution.

We show that fluorophores exhibit different performances in plant cells when fused to two interacting transcription factors. For example, mTq is well recognized as a preferred CFP variant for use as a FRET donor (Goedhart et al., 2010). In the Arabidopsis root, endodermal signal was low for SCR:mTq and undetectable for SHR:mTq (Figure 2B) relative to autofluorescence. Such low mTq detectability, however, was not reported when expressed at high levels (Figure 1C; Hecker et al., 2015) or localized to cell membranes, cytoplasm or cytoskeleton in intact Arabidopsis plants (Roppolo et al., 2011; Peremyslov et al., 2012; Waadt et al., 2014). This is possibly due to high expression levels of these fusion proteins concentrated at different subcellular domains, or might suggest that mTq protein is sensitive to the plant nuclear microenvironment. Nevertheless, our optimization procedure highlights the importance of selecting appropriate fluorophores for different cellular and subcellular conditions (see Supplementary Materials). Linker optimization between the protein-of-interest and the fluorophore is also crucial for ensuring close proximity, favorable dipole orientation and fusion protein functionality. Our studies confirmed that the linker introduced by common Gateway recombination site is sufficiently short for FRET between SHR and SCR, although functionality of N-terminal SHR fusion was only restored with shortened linker without compromising FRET detection (Figure 3). It is therefore important to optimize fusion linkers for functional *in vivo* FRET studies.

Optimizing FRET-FLIM in living Arabidopsis roots allowed visualization of spatiotemporal bindings between endogenous SHR and SCR during different developmental stages, which cannot be addressed by *in vivo* over-expressions or cell lines (Long et al., 2017). We found that the FRET levels between SYFP2-SHRΔ1a and SCR:mRFP vary among different developmental contexts, and among different cell types within each developmental stage. The enhanced FRET-FLIM signals in CEI reflect a specific conformation of a multimeric complex modified by the presence of other binding partners (Long et al., 2017). We have recently shown that SHR and SCR interact with the BIRD protein JACKDAW which regulate SHR intercellular mobility and transcriptional activity, and that SHR-SCR-JKD complexes display distinct conformations within the U-shaped domain (Long et al., 2015a, 2017). The cell cycle regulator RETINOBLASTOMA-RELATED (RBR) also physically associates with the SHR-SCR complex to repress ectopic formative divisions in the endodermis (Cruz-Ramírez et al., 2012). The *in vivo* binding dynamics of RBR and other interacting BIRD proteins to the SHR-SCR complexes have not yet been tested. To this end, extending our optimized *in vivo* FRET-FLIM technique for proteins interacting with SHR-SCR complex to create a protein interaction map at cellular resolution will be a big step toward understanding the cell-specific protein complex dynamics *in vivo* and their functions during different stages of Arabidopsis development.

The discovery of SHR-SCR interaction heterogeneity highlights the spatiotemporal sensitivity of *in vivo* FRET-FLIM. However, FRET requires the donor and acceptor being within the stringent Förster radius and the fluorophore dipoles parallel to each other, making it especially sensitive to close-ranged protein associations but inefficient to detect interactions between far-end-tagged proteins due to functionality obligations or associations of proteins within big protein complexes that exceed Förster radii. Meanwhile, single molecule spectroscopy analyses such as fluorescence correlation spectroscopy (FCS)-based techniques, can detect protein-protein association without Förster radius requirement. While single molecule tracking of SHR-SCR complex using FCS was in line with our findings (Clark et al., 2016), however, it was proven impractical in the stem cell niche due to high background level, while *in vivo* FRET-FLIM succeeded in obtaining interaction information thanks to the stringently controlled fitting procedure. To sum up, one can obtain a broader spectrum of information regarding protein-protein interaction by combining FRET-FLIM and FCS-based techniques *in vivo*.

Nevertheless, our heterologous analyses forecast future applications of *in vivo* FRET-FLIM in studying protein-protein interactions in other biological systems. Indeed, attempts of applying FRET-FLIM measurements in living animals or intact tumors to study interactions between exogenous proteins or monitor biosensors have been reported (Kelleher et al., 2009; Kardash et al., 2011; Venugopal et al., 2012; Nobis et al., 2013), promising the possibility of *in vivo* FRET-FLIM usage. Multiphoton FRET-FLIM (Peter et al., 2005) may further enhance SNR, improve detection depth in thicker tissues and reduce photobleaching, although the near-infrared excitation will likely require additional optimizations to address potential cross-excitation and signal bleedthrough for the SYFP2-mRFP pair. Following our optimization procedure, endogenous protein interactions should be readily analyzable in living animals and other multicellular organisms.

In conclusion, optimization of FRET-FLIM allows detection of protein complexes in living tissue at cellular resolution. Our optimization procedure is, in principle, appropriate for any protein interaction pair and in various subcellular compartments (Stahl et al., 2013; Somssich et al., 2015; Weidtkamp-Peters and Stahl, 2017). Additionally, homo-FRET measured by fluorescence anisotropy can help in further deciphering protein complex compositions. Low abundance of certain proteins and potential limitations in engineering effective fusions without disrupting protein function still remain as major challenges for *in vivo* FRET-FLIM measurements. Technical advances will rely on continuous improvements of fluorescent tags and detection sensitivity. Characterizing and implementing mTurquoise2, mScarlet (Bindels et al., 2017) and other fluorophores with high quantum yield in future FRET-FLIM measurements, in addition to the application of other microscopic techniques such as single-molecule FRET-FLIM or FCS-based techniques in living organisms, will allow us to precisely monitor the composition of multiprotein complexes and their dynamics *in vivo*.

## Author contributions

The scientific conception, is due to IB and YL. IB and YL designed and executed the experiments. YS, SW-P assisted in setting up, optimizing FRET-FLIM experiments and data analysis. JG helped with FRET-FLIM measurements in protoplast. TG helped with discussions related to FRET-FLIM quantification. IB and YL wrote the manuscript. All authors were involved in data analysis, interpretation and revision of the manuscript.

### Conflict of interest statement

The authors declare that the research was conducted in the absence of any commercial or financial relationships that could be construed as a potential conflict of interest.
