# Supplementary material for: Optimizing FRET-FLIM Labeling Conditions to Detect Nuclear Protein Interactions at Native Expression Levels in Living Arabidopsis Roots
Source: Front Plant Sci. 2018 May 15;9:639. doi: 10.3389/fpls.2018.00639 (PMC5962846; doi:10.3389/fpls.2018.00639)
Supplement: Supplementary file 1 [file Data_Sheet_1.PDF]

Supplementary Material

Considerations to take into account using FRET-FLIM

FRET-FLIM in transient system "ex-in vivo FRET-FLIM"

Generally FRET-FLIM measurements are done with high expression levels. Here the choice of the fluorophore is not critical but rather the position of the N or C terminal of the interacting protein pairs which determines the FRET-FLIM occurrence and efficiency.

FRET-FLIM in living tissues "in vivo FRET-FLIM"

In addition to the criteria cited above, the following considerations should be taken into account.

1- Direct interaction of the protein pairs: FRET measures the physical proximity of interacting proteins. Thus, prior to proceeding the study in living plant tissues, direct interaction should be checked to avoid influence of plant specific factors. Tests can be performed using an in vitro heterologous system such as the yeast two hybrid, split luciferase in mammalian cells or co-immunoprecipitation using recombinant protein expression in bacteria.

2- Expression levels: the promoter expression level should be sufficiently high (can be judged by signal-to-background ratio, this can vary extremely, depending on the specific tissue). If this condition is not met, a promoter with higher expression in the same tissue but without introducing phenotypes (e.g. altered tissue structure, altered response or sensitivity to tested conditions) can be used instead.

3- Functionality of the fusion: When expressed under endogenous or tissue specific promoter, functionality of the resulting fusion protein should be checked by assessing the level of expression, correct subcellular localization, no additional phenotype and mutant complementation.

General workflow

Prior to in vivo test

- Establish or confirm interaction between your protein-pair-of-interest in an in vitro or ex vivo system (yeast two hybrid, transient bimolecular fluorescence/luciferase complementation, co-immunoprecipitation, etc.).
- Test FRET efficiency between your protein pair with alternated fluorophore tagging orientations in a transient system (e.g. mesophyll protoplast), and select the combination with highest FRET efficiency. Note that certain proteins (e.g. transmembrane proteins) might require alternative tagging positions.

In vivo optimization

- Independently test whether the expression level of your protein pair is high enough to conduct FRET-FLIM by assessing signal-to-background ratio. Test with intended donor tags to ensure sufficient FLIM measurement. Note the potential effect of background components with high auto-fluorescence (e.g. chlorophyll).

- Choose the right fluorophores depending on their performance (signal-to-background ratio) under endogenous promoters.
- Check functionality of your selected fusion combination from in vitro / ex vivo tests by complementing mutants. If modifications of fusion proteins are needed, re-check ex vivo FRET efficiency and in vivo expression level as mentioned above.

Specific

- Use young and healthy plants since some interactions are dependent on the physiological conditions.
- Tissue should be mounted either in water or in established mounting medium with limited auto-fluorescence on a slide and use a cover slip with a thickness matching the objective lens, usually 0.17 mm (# 1.5) thickness. This has important influence on the sensitivity of the optical system.

Pitfalls/cautions

- Make sure that the measurements are done using the same confocal set up: laser power, pinhole, number of frames acquired, and focal plane.
- Very often, samples are moving out of the focal plane either because: 1- excess of water in the preparation, 2- focal drift (in that case try using a confocal equipped with an incubation chamber to keep temperature stable).
- Saturation / overexposure on photon counting detector can lead to artefacts of seemingly reduced lifetime (pile-up effect), which could potentially lead to false positive reading of FRET.
- Meanwhile, low photon count may cause more low-lifetime background contribution, thereby also leading to false positive reading of FRET. Therefore, photon count range should be strictly checked, commonly at least 200 photons per pixel, over 1000 photons is advisable. If you have low expression, rather increase the frame number to accumulate more photons than increase laser power.
- Laser power at the objective should be checked and not exceed 1-2 µW, as more laser power will lead to bleaching artefacts.

Comment problems

Step	Problem	Possible reason	Solution
Testing interaction in transient system	Low FRET efficiency	-wrong position of the tag<br>-proteins not interacting directly	-Check N and C terminal tagging<br>-Test interaction using in vitro system.
Image acquisition	Samples out of focus during acquisition	-sample is mounted in excess of water.<br>-Focal drift due to changes in focal plane due to changes in temperature or air currents and vibrations within the room	-remove water excess.<br>- Control the temperature and isolate the stage using an incubating chamber.<br>-Prevent vibrations by using an anti-vibration or air table.
Scanning	Signal getting lower while scanning	Bleaching due to too high laser power	Adjust laser power intensity with a Laser Power Meter

Software used for FRET-FLIM analysis.

The software used for this study can be downloaded/requested from: http://www.mpc.hhu.de/software/software-package.html.

If data are acquired using the TCSPC electronics from PicoQuant then analysis could be performed using SymPhoTime 64 Fluorescence Lifetime Imaging and Correlation Software commercially available and user friendly for data analysis (https://www.picoquant.com/products/category/software).

Supplementary Table 1. Primer list for cloning

SCFP3A/mTurquoise-R2R3 F	5’-GGGGACAGCTTTCTTGTACAAAGTGGCTATGGTGAGCAAGGGCGAGGAG-3’
SCFP3A/mTurquoise-R2R3 R	5’-GGGGACAACTTTGTATAATAAAGTTGCTTACTTGTACAGCTCGTCCATGC-3’
SCFP3A/mTurquoise-221 F	5’-GGGGACAAGTTTGTACAAAAAAGCAGGCTTCATGGTGAGCAAGGGCGAGGAG-3’
SCFP3A/mTurquoise-221 R	5’-GGGGACCACTTTGTACAAGAAAGCTGGGTCCTTGTACAGCTCGTCCATGC-3’
SYFP2-R2R3 F	5’-GGGGACAGCTTTCTTGTACAAAGTGGCTATGGTGAGCAAG-3’
SYFP2-R2R3 R	5’-GGGGACAACTTTGTATAATAAAGTTGCTCACTTGTACAGC-3’
SYFP2-221 F	5’-GGGGACAAGTTTGTACAAAAAAGCAGGCTTCATGGTGAGC-3’
SYFP2-221 R	5’-GGGGACCACTTTGTACAAGAAAGCTGGGTCTCACTTGTACAGC-3’
mCherry/mStrawberry-R2R3 F	5’-GGGGACAGCTTTCTTGTACAAAGTGGCTATGGTGAGCAAGGGCGAGGAG-3’
mCherry/mStrawberry-R2R3 R	5’-GGGGACAACTTTGTATAATAAAGTTGCTTACTCACTTGTACAGCTCGTCCATGCC-3’
mCherry/mStrawberry-221 F	5’-GGGGACAAGTTTGTACAAAAAAGCAGGCTGTATGGTGAGCAAGGGCGAGGAG-3’
mCherry/mStrawberry-221 R	5’-GGGGACCACTTTGTACAAGAAAGCTGGGTTCTTGTACAGCTCGTCCATGCCG-3’
mRFP-R2R3 F	5’-GGGGACAGCTTTCTTGTACAAAGTGGCTATGGCCTCCTCCGAGGACGTCATC-3’
mRFP-R2R3 R	5’-GGGGACAACTTTGTATAATAAAGTTGCTCAGGCGCCGGTGGAGTGGCGGCCC-3’
mRFP-221 F	5’-GGGGACAAGTTTGTACAAAAAAGCAGGCTGTATGGCCTCCTCCGAGGACGTC-3’
mRFP-221 R	5’-GGGGACCACTTTGTACAAGAAAGCTGGGTTGGCGCCGGTGGAGTGGCGG-3’
NLS 5’-attachment F	5’-ATGCCAAAGAAGAAGAGAAAGGTCATGGTGAGCAAGGGCG-3’
NLS-221 F	5’-GGGGACAAGTTTGTACAAAAAAGCAGGCTTCATGCCAAAG-3’
SHR-R2R3 F	5’-GGGGACAGCTTTCTTGTACAAAGTGGCTATGGATACTCTCTTTAGACTAGTCAGTCTCC-3’
SHR-R2R3 R	5’-GGGGACAACTTTGTATAATAAAGTTGCTTACGTTGGCCGCCACGCACTAGCCCAAAC-3’
SCR-R2R3 F	5’-GGGGACAGCTTTCTTGTACAAAGTGGCTATGGCGGAATCCGGCGATTTCAACGGTGG-3’
SCR-R2R3 R	5’-GGGGACAACTTTGTATAATAAAGTTGCCTAAGAACGAGGCGTCCAAGCTGAAGC-3’
SYFP2-SHRΔ1a sense	5’-GACGAGCTGTACAAGGACAAAGTGGCTATGGATACT-3’
SYFP2-SHRΔ1a antisense	5’-AGTATCCATAGCCACTTTGTCCTTGTACAGCTCGTC-3’
SYFP2-SHR sense	5’-CATGGACGAGCTGTACAAGATGGATACTCTCTTTAGAC-3’
SYFP2-SHR antisense	5’-GTCTAAAGAGAGTATCCATCTTGTACAGCTCGTCCATG-3’
SCR-mCherryΔ1a sense	5’-GGACGCCTCGTTCTAACAAAGTGGCTATGGTGAG-3’
SCR-mCherryΔ1a antisense	5’-CTCACCATAGCCACTTTGTTAGAACGAGGCGTCC-3’
