# Supplementary material for: Optimizing FRET-FLIM Labeling Conditions to Detect Nuclear Protein Interactions at Native Expression Levels in Living Arabidopsis Roots
Source: Front Plant Sci. 2018 May 15;9:639. doi: 10.3389/fpls.2018.00639 (PMC5962846; doi:10.3389/fpls.2018.00639)
Supplement: Supplementary file 2 [file Image_1.pdf]

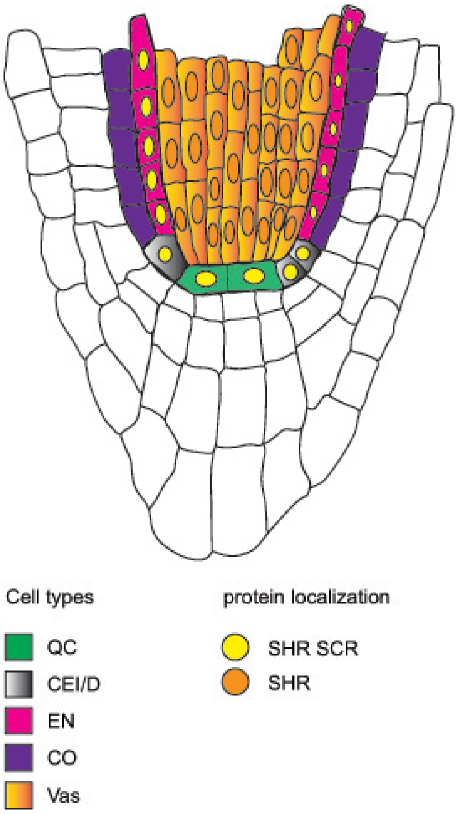

Supplementary Figure 1: Diagram of *Arabidopsis thaliana* root meristem.

Arabidopsis thaliana root meristem marked with different cell types. QC, quiescent center; CEI, cortex-endodermal initial. Right half panel summarizes SHR and SCR localization.

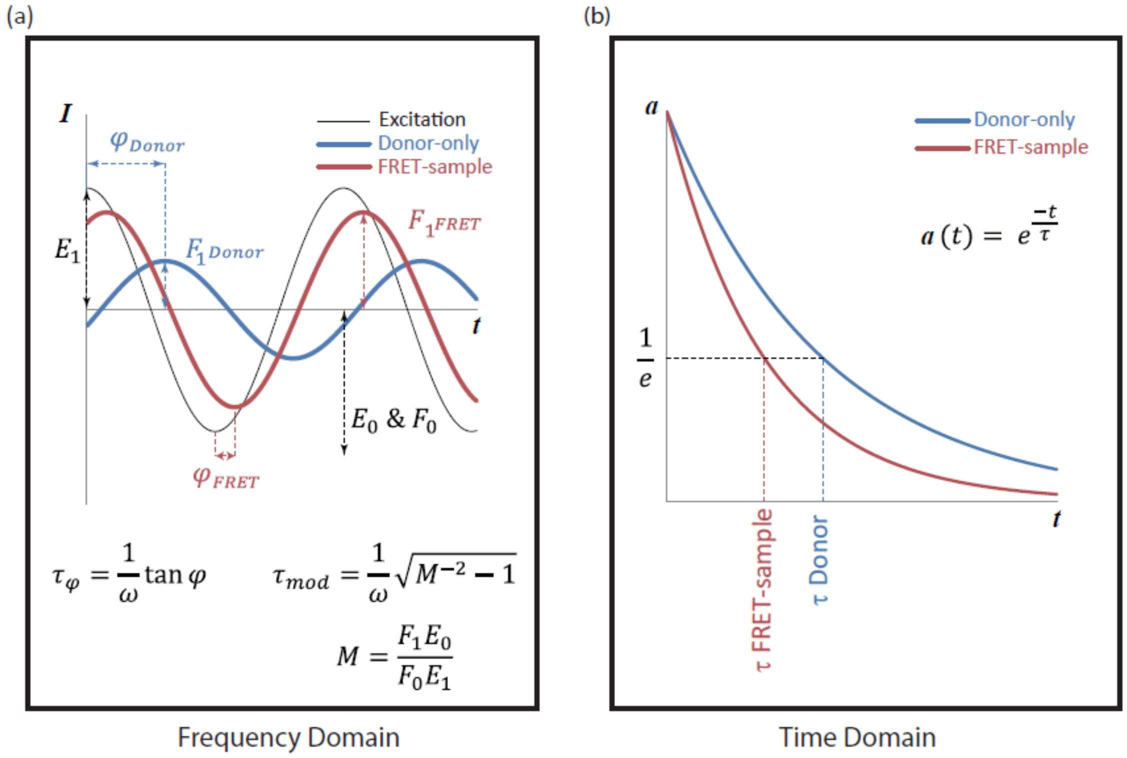

Supplementary Figure 2

Schematic representations of the time domain and frequency domain for fluorescence lifetimes determination.

(a) Frequency-domain FLIM resolves lifetime from the phase shift and modulation of the fluorescence emission. *I*, fluorescence intensity (co-aligned); φ, phase shift; ω, laser angular frequency, *M*, modulation ratio; *E*, Excitation; *F*, Emission. In the frequency domain the life times are represented by two values, (τ_{φ}) derived from the phase angle (φ_{ω}) and (τ_{mod}) determined from the modulation. The τ_{φ} and τ_{mod} values are usually strongly correlated and each value represents a different weighted average of the decay times displayed by the sample. (b) Time-domain FLIM relies on single-photon counting, molecules are excited by a very short pulse. The fluorescence life time lifetime is based on the exponential decay of fluorescence signal measured by Time-Correlated Single Photon Counting (TCSPC). *a*, normalized photon count; *t*, time; τ, lifetime;

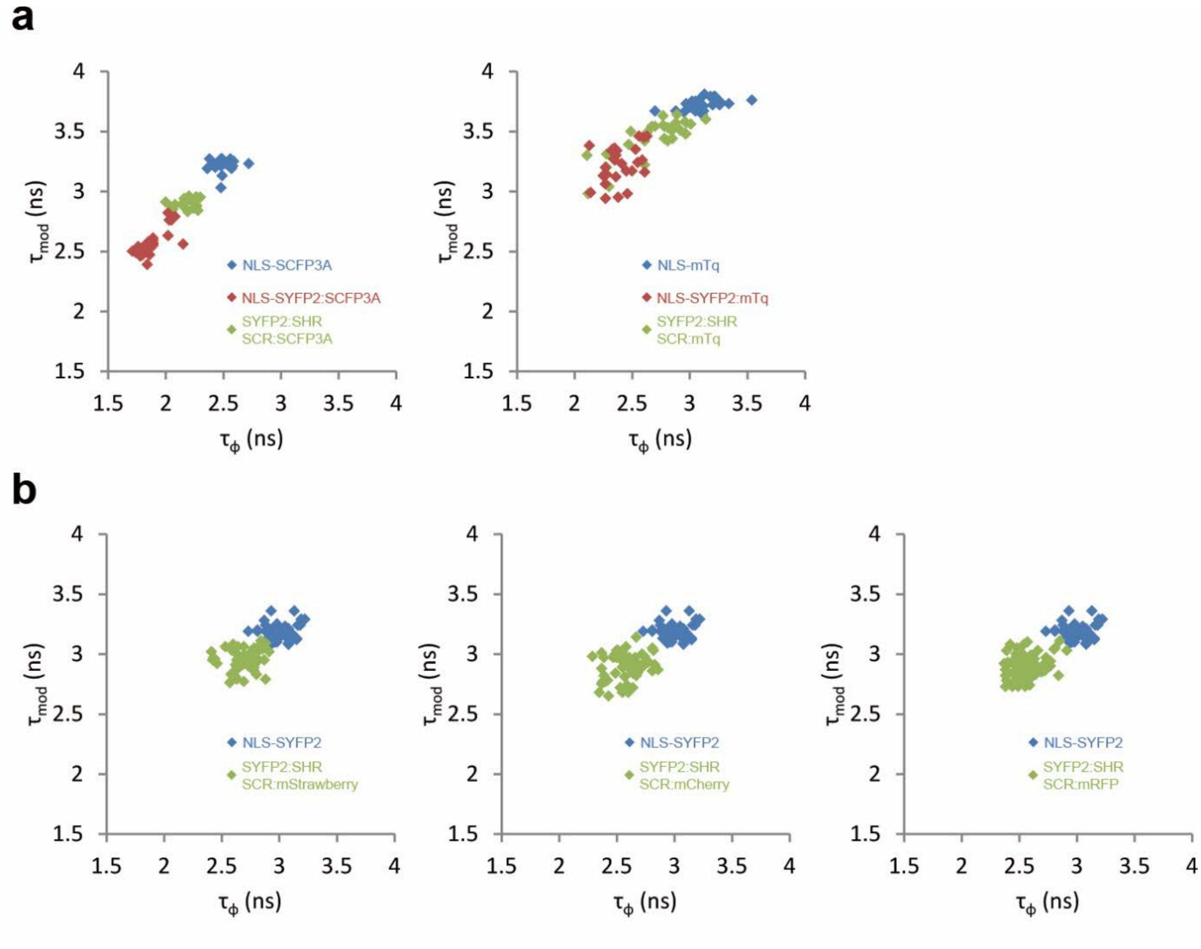

Supplementary Figure 3: Optimization of tags-of-choice for FRET-FLIM between SHR and SCR.

(a) Scatterplots showing distribution of phase lifetime τ_φ against modulation lifetime τ_{mod} from protoplast measurements between SYFP2:SHR and SCR:SCFP3A or SCR:mTq. (b) Scatterplots of fluorescence lifetime measurements between SYFP2:SHR and SCR:mStrawberry, SCR:mCherry or SCR:mRFP in protoplasts.

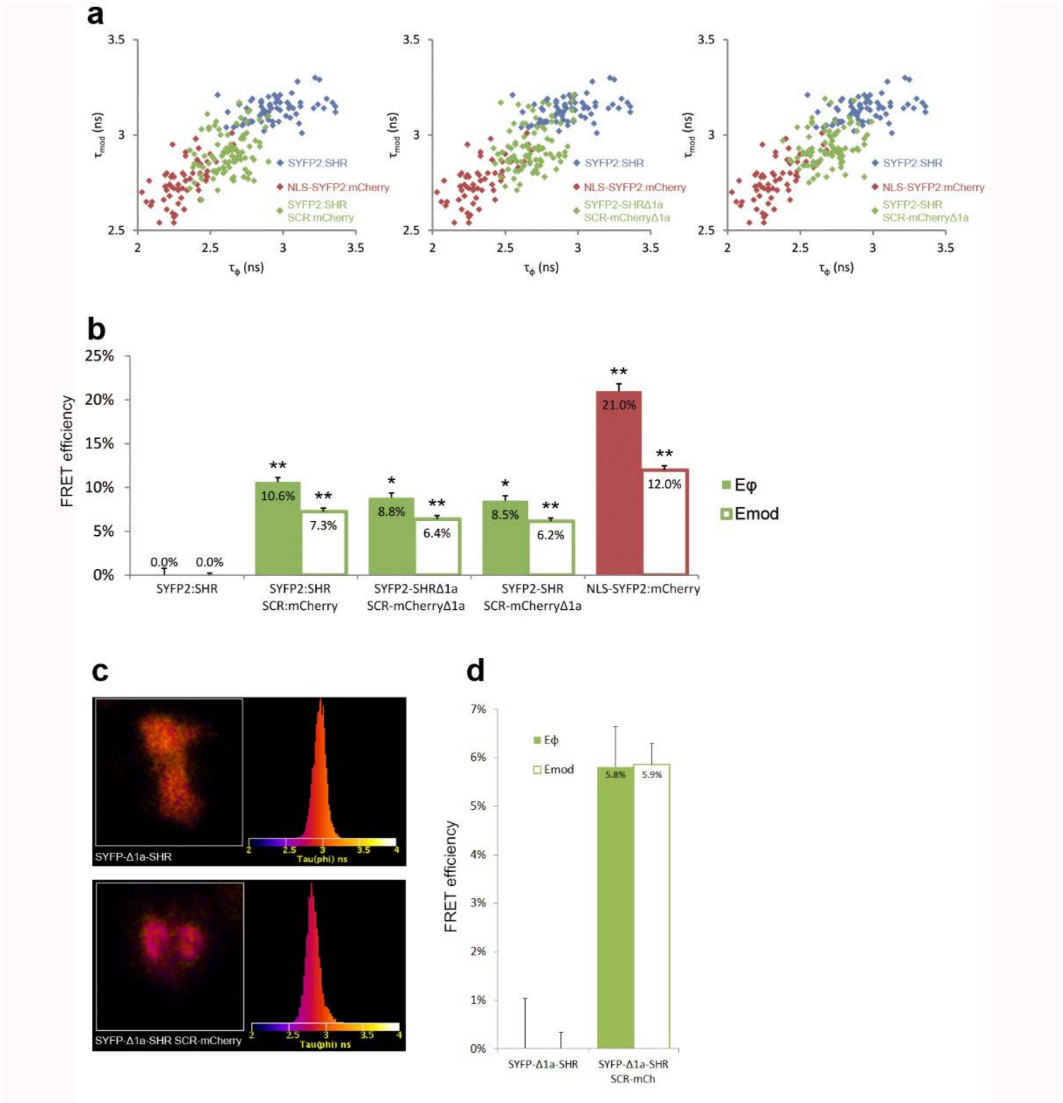

Supplementary Figure 4: Optimization of linker composition for FRET-FLIM between SHR and SCR.

(a) Scatterplot of fluorescence lifetime measurements between SHR and SCR with different linker composition in protoplasts. SYFP2-SHRSΔ1a has DKVA linker, SCR-mCherryΔ1a has NKVA linker, while SYFP2-SHR has no linker between SYFP2 and SHR. (b) Bar chart of FRET efficiency *E* derived from (a), with error bars of standard error of mean. *, $10^{-20} < p < 10^{-2}$; **, $p < 10^{-20}$. (c) Fluorescence lifetime heatmaps and histograms of SYFP-Δ1a-SHR in donor-only or FRET sample HeLa cells. (d) Bar chart showing FRET efficiency *E* derived from τ_{φ} and τ_{mod} in (c), error bars represent standard errors within one set of experiment, n > 10.
